# Incidence and Survival of Thoracic Angiosarcoma: Epidemiologic Evidence from a Population-Based Cancer Registry

**DOI:** 10.3390/cancers18040612

**Published:** 2026-02-13

**Authors:** Niels Michael Dörr-Jerat, Ina Wellmann, Franziska Rees, Marcus Krüger, Hiltraud Kajüter, Andreas Stang

**Affiliations:** 1 Department of Thoracic Surgery, Martha-Maria Hospital Halle-Dölau, 06120 Halle, Germany; 2 University Hospital Halle, Medical Faculty of the Martin Luther University Halle-Wittenberg, 06120 Halle, Germany; 3 Cancer Registry of North Rhine-Westphalia, 44801 Bochum, Germany; 4 Institute of Medical Informatics, Biometry and Epidemiology, University Hospital Essen, 45147 Essen, Germany

**Keywords:** rare malignancy, angiosarcoma, thorax, incidence, survival

## Abstract

Introduction: Thoracic angiosarcoma is a very rare and malignant disease. This study examined the incidence and survival of thoracic angiosarcomas with special focus on primary and secondary angiosarcomas. Methods: Data from the population-based North Rhine-Westphalia (NRW) cancer registry in Germany covering the years 2008–2023 were analyzed, including primary and secondary thoracic angiosarcoma (ICD-O-3: morphology 9120/3, topography C34, C38, C44.51, C49.3, C50), and reported age-standardized incidence rates (using the Old European Standard population) and survival analyses (Kaplan–Meier curves). Results: A total of 421 cases of thoracic angiosarcoma were analyzed, and 90.0% of the patients were female (*n* = 379). Age-standardized incidence rates of thoracic angiosarcoma were 0.25 per million per year for male patients (SE 0.0) and 1.5 per million per year for female patients (SE 0.1). All male patients had primary angiosarcoma (*n* = 42). Among female patients, the majority of cases were second primary tumors (*n* = 262, 69.1%). The 5-year overall survival (OS) for the cohort study was 38.5% (SE 2.6). Stratified by sex, OS was 41.1% (SE 2.8) for women and 12.0% (SE 5.4) for men. Among female patients, OS was 40.9% (SE 4.1) for primary and 41.8% (SE 3.8) for second primary angiosarcoma. The worst OS occurred among patients with angiosarcoma of the lung (men 20.0% (SE 12.7)) and of the mediastinum, heart and pleura (men 4.7% (SE 4.5)) The OS for women was 0%; all females died within 2.2 years after diagnosis of angiosarcoma with these topographies. Conclusions: Thoracic angiosarcoma is a rare condition with a poor prognosis. Irrespective of whether their disease is classified as primary or second primary, women with angiosarcoma have a better prognosis than men. Topography seems to be the most determining prognostic factor in this disease.

## Simple Summary

Thoracic angiosarcoma is a very rare, highly aggressive malignant vascular tumor that may involve the lung, breast, chest wall, heart, or adjacent thoracic tissues. Using data from a large population-based cancer registry in Germany, we analyzed the incidence and post-diagnosis survival of this disease. A markedly higher incidence of thoracic angiosarcoma was observed in women than in men, largely attributable to cases arising as secondary malignancies following prior cancer treatment, with longer survival observed in women than in men for both primary and second primary angiosarcomas. Survival was strongly dependent on tumor location, with particularly poor outcomes observed for angiosarcoma of the lung, heart, mediastinum, or pleura. These findings highlight the aggressive nature of thoracic angiosarcoma and the importance of tumor location in determining patient prognosis.

## 1. Introduction

Angiosarcoma is a rare disease that accounts for 1–2% of soft tissue sarcomas. It arises from endothelial cells of small blood and lymphatic vessels and can occur in any part of the body [1,2]. Cutaneous angiosarcomas are the most common subtype, affecting the skin, breast, and soft tissues of the head and neck, particularly the scalp [2]. Angiosarcomas often occur as so-called second primary cancers after radiotherapy for breast cancer or skin cancer [3]. There is also an association with chronic lymphedema following lymph node dissection for treatment of breast cancer or malignant melanoma, a condition known as Stewart–Treves syndrome [4,5]. The association between malignant melanoma and thoracic angiosarcoma is less well established [6] than the association with breast cancer [2]. However, it is not the malignancy itself that leads to the development of angiosarcoma, but rather the therapeutic consequences—such as radiotherapy and chronic lymphedema—which are common to these cancers. The most studied and common causes of second primary angiosarcoma are previous cancer with subsequent radiation and/or chronic lymphedema [7,8]. Additional proposed risk factors include ultraviolet radiation, immunocompromised states, arteriovenous fistulas, xeroderma pigmentosum, trauma, foreign bodies, thorium dioxide exposure, and viral infections [9,10,11,12]. It is therefore important to distinguish between spontaneous primary angiosarcomas and second primary angiosarcomas. Clinical experience and published studies indicate that the latency period between the primary malignancy and the development of angiosarcoma is approximately 5–10 years, with the strongest evidence coming from breast cancer research [5,7,13,14]. In most cases, causal factors appear to be unknown or very difficult to assess. For example, in 1999, Maziak et al. reported a case of angiosarcoma developing in a thoracotomy incision 17 years after surgery for UICC stage I lung cancer [9].

In general, thoracic angiosarcomas often present with nonspecific or seemingly benign symptoms despite the presence of advanced disease with infiltrative growth and metastatic spread [10]. For example, in patients with tumors of pleural origin, the most common symptoms include dyspnoea, chest tightness, pain, pleural thickening, pleural effusion and recurrent hemothoraces. However, symptoms vary depending on the tumor location. Diagnosis can be difficult, even with a valid biopsy, and misdiagnosis is possible [15]. Mimicry of other rare angiogenic malignancies, such as hemangioendothelioma or intimasarcoma, and even benign tumors is possible. Surgery is indicated for localized tumors, but the prognosis remains poor. Although most patients die shortly after diagnosis, some authors suggest that a multidisciplinary approach including surgery, radiotherapy and chemotherapy may result in prolonged survival [10].

Thoracic angiosarcoma is an extremely rare malignancy. The breast and chest wall are the most frequent locations. Banks et al. published the results of a cohort of 183 patients with 34 primary and 149 second primary angiosarcomas of the breast and chest wall [16]. Other sites are even rarer. According to Wang et al., about 43 cases of pleural angiosarcomas have been reported on PubMed up to 2022 [17]. In the case of cardiac angiosarcoma, only small institutional series and literature reviews are available, typically ranging from single case reports to institutional experiences of 47–122 cases accumulated over several decades [18,19,20].

The most recent comprehensive epidemiological data on angiosarcoma were published by Wagner et al., 2024 [3]. Their study included 19,289 patients in the USA who received a new diagnosis of angiosarcoma between 2001 and 2020, as recorded in the U.S. Cancer Statistics database. In that analysis, angiosarcomas of the chest wall and thorax were classified within the cutaneous, subcutaneous and breast angiosarcoma subgroup, whereas angiosarcomas of the heart, trachea, lung, pleura and mediastinum were categorized into the visceral angiosarcoma subgroup [3]. According to Sturm et al., 5-year overall survival (OS) ranges from 30% to 56% [8]. In population-based cohorts, the 5-year overall survival rate for angiosarcoma is approximately 26–30%, with substantial variation according to anatomic site, disease stage, and whether the tumor is primary or secondary [21,22,23].

Although these publications provide relevant epidemiological findings on angiosarcoma, their applicability to thoracic angiosarcoma is limited. To date, no distinction has been made in the literature between primary and second primary angiosarcomas or according to tumor topography. Furthermore, site-specific survival analyses were not reported by Wagner et al. Consequently, epidemiological evidence on thoracic angiosarcoma, including its distribution across anatomical sites, remains limited. The aim of this study is to provide population-based age-standardized incidence rates and 5-year OS for thoracic angiosarcomas between 2008 and 2023 in North Rhine-Westphalia, Germany.

## 2. Materials and Methods

The Cancer Registry of the Federal State of North Rhine-Westphalia (LKR NRW) covers a population of 18 million people. Cancer reporting is mandatory, and the completeness of cancer registration is evaluated regularly by the German Centre for Cancer Registry Data at the Robert Koch-Institute. Since 2008, data of good quality have been available for North Rhine-Westphalia. Patients diagnosed with thoracic angiosarcoma between 2008 and 2023 were included in the study. New cases of thoracic angiosarcoma were identified using the International Classification of Diseases for Oncology, Third Edition (ICD-O-3) based on morphology (9120/3 hemangiosarcoma) and topography codes (C34 bronchus and lung, C38 heart, mediastinum and pleura, C44.51 skin of thorax, C49.3 soft tissues of thorax, C50 breast). Mortality follow-up for cancer patients was routinely assessed by the LKR NRW through a passive mortality-follow-up using electronic reports of all deceased individuals in NRW obtained from population registration offices.

Age-standardized incidence rates by sex were calculated for the study period using the Old European Standard population [24]. The median survival was computed as the shortest time at which the survival probability dropped to 0.5 or below. Brookmeyer and Crowley constructed the confidence interval for the median survival [25].

The Kaplan–Meier method was applied to estimate 5-year overall survival [26]. Censoring occurred at the last date of mortality follow-up, which was 31 December 2023. Time-to-event data were analyzed using Cox proportional hazards regression to estimate the hazard ratios and 95% confidence intervals. Survival analyses were stratified according to primary versus second primary thoracic angiosarcoma (i.e., an angiosarcoma developing after a previous diagnosis of breast or skin cancer) and by tumor topography.

In light of the frequent association between angiosarcoma and prior malignant melanoma and/or breast cancer, patients were stratified into two subgroups: those with a history of breast cancer and/or malignant melanoma, classified as *second primary angiosarcoma*, and those without evidence of previous breast cancer or malignant melanoma, classified as *primary angiosarcoma*. Secondary cancer, also known as metastatic cancer, is cancer that has spread from one part of the body to another. In contrast, *second primary cancer* is a completely new malignancy in a person who has already had cancer. It can occur in the same organ or in a different part of the body. It is sometimes caused by a side effect of previous radiation therapy or chemotherapy.

Based on clinical evidence and the literature [7,8], the latency between the diagnosis of the previous malignancy and the development of angiosarcoma was categorized into two intervals: 0–5 years and >5 years. Further subgroup analysis demonstrated that the majority of cutaneous malignancies were basal cell carcinomas (*n* = 32). These cases were excluded from the analysis because no established association with angiosarcoma has been reported in the literature and, furthermore, because radiation or chronic lymphedema are not associated with basal cell carcinoma.

## 3. Results

The cancer registry recorded a total of 421 cases of hemangiosarcoma (hereafter referred to as angiosarcoma) between 2008 and 2023. Of these cases, 10% occurred in male patients (*n* = 42) and 90% in female patients (*n* = 379). The most frequent anatomical sites were the breast (*n* = 250) and the soft tissue of the thorax (*n* = 86). However, 20.2% of all angiosarcomas were located at anatomical sites other than the breast and thoracic soft tissue (Table 1).

The median age at diagnosis of all patients was 69.1 years (standard error, SE 14.3). While the median age at diagnosis for women was 70.8 years (SE 12.5), men were diagnosed at an earlier median age of 54.4 years (SE 19.8) (Table 1).

**Figure 1:**
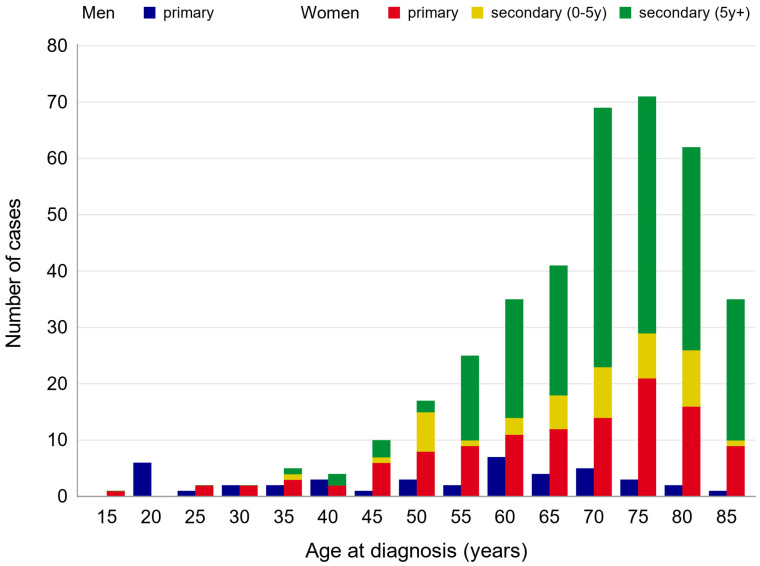
Age distribution of newly diagnosed male and female primary and second primary angiosarcoma in North Rhine-Westphalia, 2008–2023.

Between 2008 and 2023, the age-standardized incidence of thoracic angiosarcoma ranged from 0.00 to 0.70 per million per year in male patients and ranged from 1.0 to 2.3 per million per year in female patients (Figure 1).

**Figure 2:**
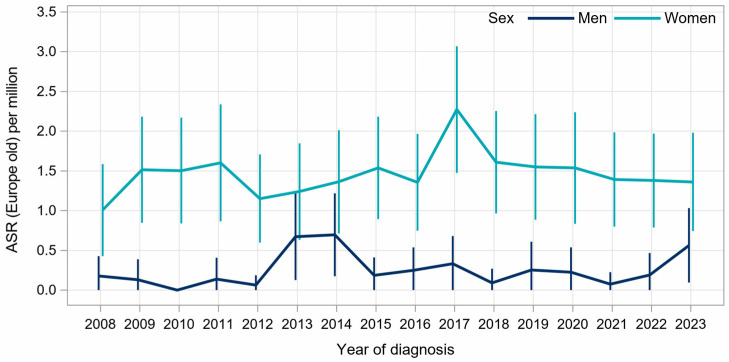
Age-standardized annual incidence rates (ASR, cases per million person-years) of thoracic angiosarcoma in North Rhine-Westphalia, 2008–2023.

A total of 67.7% of patients (*n* = 285) had experienced at least one prior thoracic cancer diagnosis before the development of thoracic angiosarcoma. Breast cancer (*n* = 262) and skin cancers (*n* = 38) were the most frequently observed malignancies. Among all patients with thoracic angiosarcoma, 51.1% had a history of breast cancer or malignant melanoma more than 5 years prior to the diagnosis of angiosarcoma. Considering these conditions (breast and/or skin cancer with a latency of more than 5 years), all 42 male patients were classified as having primary angiosarcoma. In contrast, 56.7% of female patients experienced previous malignant melanoma and/or breast cancer >5 years before angiosarcoma (*n* = 215) and 12.4% within the last 5 years preceding angiosarcoma (*n* = 47), while 30.9% had primary thoracic angiosarcoma (*n* = 117) (Figure 2).

**Figure 3:**
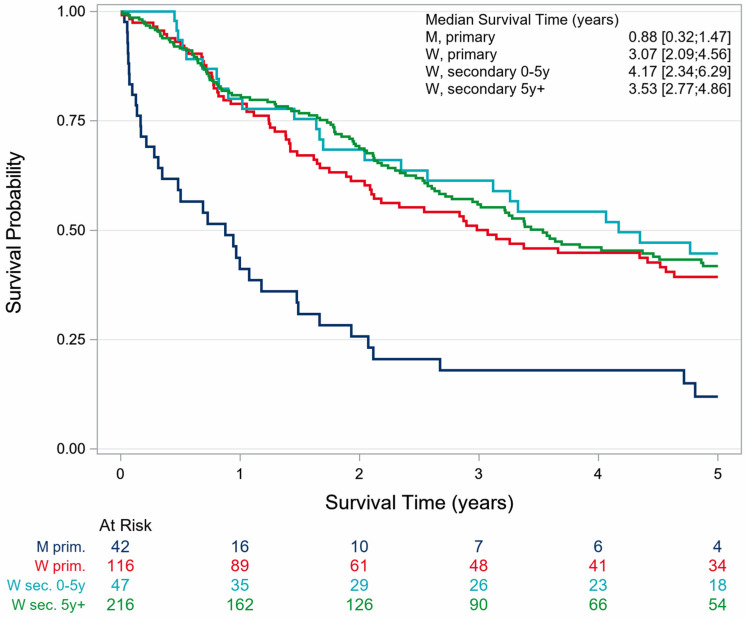
Sex-specific survival of patients with thoracic angiosarcoma stratified by a previous skin and/or breast cancer history in North Rhine-Westphalia, 2008–2023.

The 5-year OS for patients diagnosed with angiosarcoma was 38.5% (SE 2.6). The OS was 41.4% (SE 2.79) for women and 12.0% (SE 5.4) for men. The OS for female patients with primary angiosarcoma and with second primary angiosarcoma was 40.9% (SE 4.1) and 41.8% (SE 3.8), respectively (Table 2, Figure 3).

**Figure 4:**
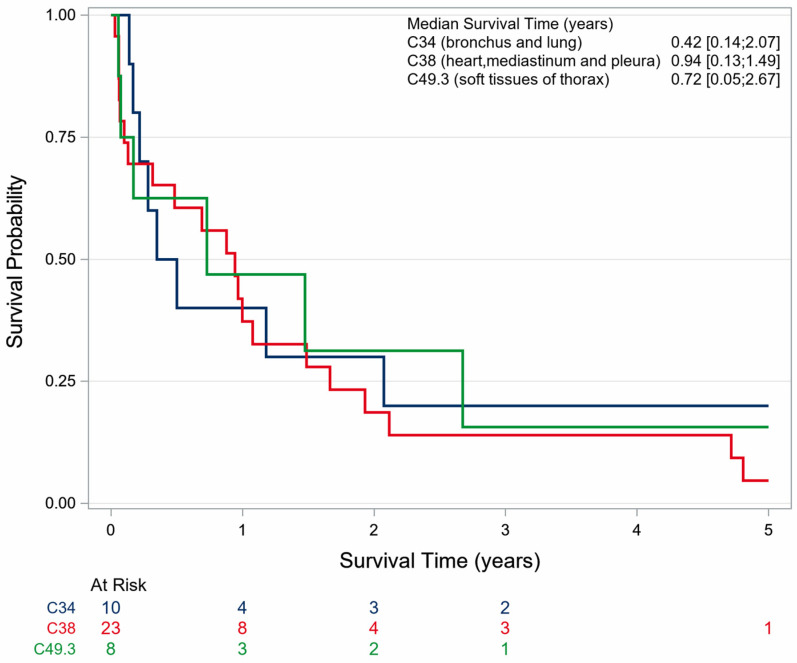
Survival of male patients with thoracic angiosarcoma stratified by topography in North Rhine-Westphalia, 2008–2023.

**Figure 5:**
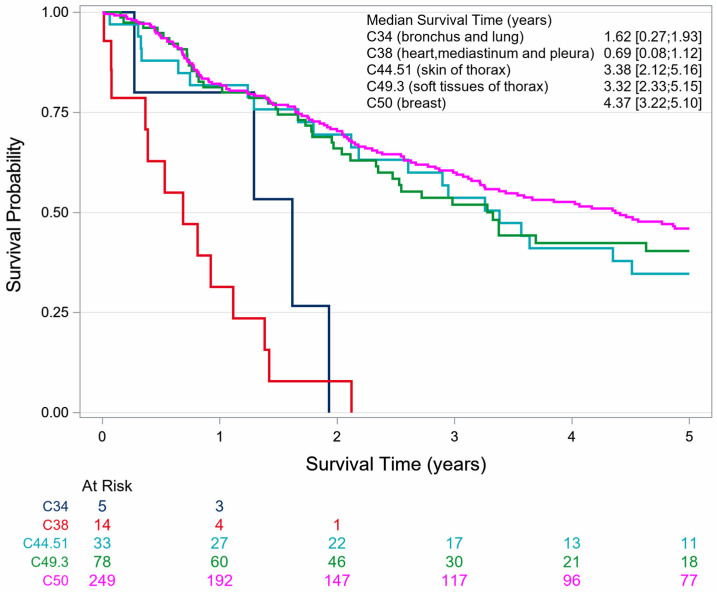
Survival of female patients with thoracic angiosarcoma stratified by topography in North Rhine-Westphalia, 2008–2023.

**Figure 6:**
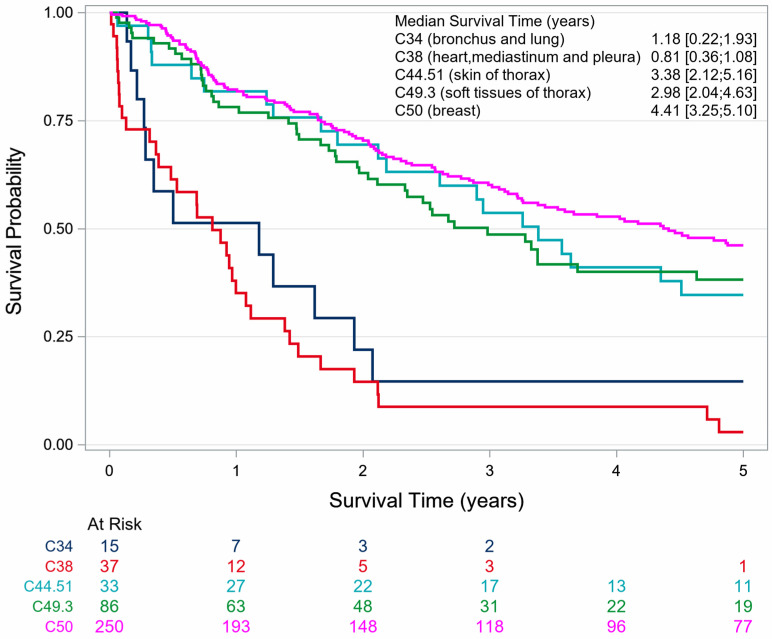
Survival of patients with thoracic angiosarcoma stratified by topography in North Rhine-Westphalia, 2008–2023.

For women and men together, it appears that angiosarcomas of the lung (C34) and angiosarcomas of the pleura, heart and mediastinum (C38) have a markedly poorer OS than all other topographies. Female patients with angiosarcoma of these topographies have a poor prognosis; their 5-year OS was 0%. All females died within 2.2 years after diagnosis of angiosarcoma (C34 and C38), which indicates a worse OS than observed in male patients (Table 2, Figure 4, Figure 5 and Figure 6).

**Figure 7:**
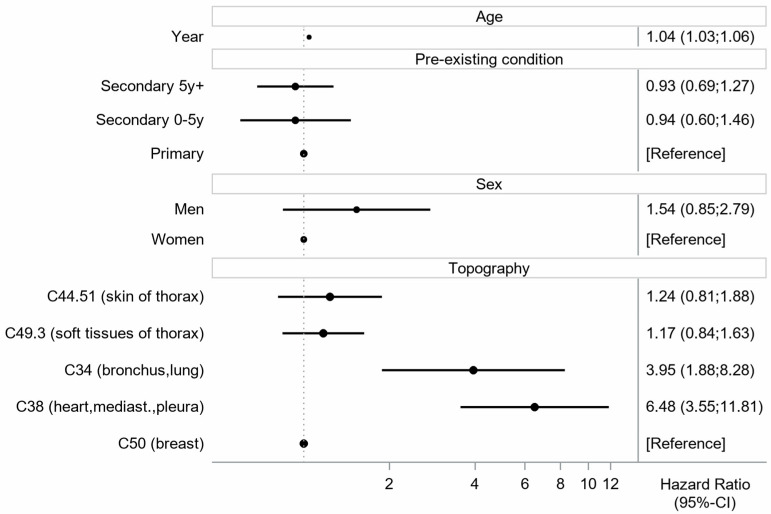
Results of the Cox proportional hazard regression with covariates age, pre-existing condition, sex and topography.

The Cox proportional hazard regression confirmed the results of the Kaplan–Meier approach. Patients with topography C38 (heart, mediastinum, pleura) showed a hazard of 6.48 (95%-CI 3.55–11.81), and for those with topography C34 (bronchus, lung), the hazard was 3.95 (95%-CI 1.88–8.28) compared with the reference topography C50 (breast). The hazard for age increases about 4% per year. This corresponds to an increase of about 50 percent after 10 years of age (Figure 7).

## 4. Discussion

A total of 421 patients with angiosarcoma were included in the analysis from the largest German population-based registry. Women were affected at least four times more often than men and had a median age at diagnosis about 15 years greater than that of men. In the U.S., 54.5% of all angiosarcomas are reported in women, and within the thoracic subgroup, angiosarcoma is observed more often in older women [3]. Although the overall prevalence is higher in women, cases of angiosarcoma in young men have also been documented [27,28,29]. This predominance in women may, in part, be explained by the well-established association between breast cancer, its treatment, and the subsequent development of angiosarcoma. Consequently, a higher frequency of angiosarcoma in women compared with men can be expected. Breast cancer is reported to occur approximately 100 times more frequently in women than in men [27,30].

Almost 70% of female patients had a second primary angiosarcoma, which was associated with a history of prior cancers. In the present study, no male patients were observed with a second primary angiosarcoma. Therefore, we emphasize that this represents an absolute rarity in men with thoracic angiosarcoma.

It was assumed that all patients with angiosarcomas for whom no other cancers had been reported to the cancer registry prior to the diagnosis of angiosarcoma had primary angiosarcomas. As the history of cancer cases could only be traced back to 2008, the proportion of second tumors is likely underestimated, since other primary cancers may have occurred before this date. Furthermore, the distinction between primary and second primary angiosarcoma remains vague due to the often difficult-to-determine relationship between tumors.

A 5-year overall survival (OS) of 38.5% was observed. Interestingly, the prognosis for women with angiosarcoma was markedly better than that for men. Even among patients with primary angiosarcoma, women demonstrated a superior prognosis compared with men. Only 12.0% of men were still alive 5 years after the diagnosis of thoracic angiosarcoma, while about 41.4% of all women were still alive after 5 years.

In the present study, no decisive differences in outcomes between primary and secondary angiosarcoma were found. In 2023, Chau et al. identified a trend of increasing incidence of second primary breast angiosarcoma while the incidence of primary breast angiosarcoma remained stable [31]. Multiple large cohort studies and meta-analyses have demonstrated that primary angiosarcoma is associated with poorer overall survival and higher disease-specific mortality compared to secondary angiosarcoma. In population-based studies of histologically confirmed angiosarcoma, the median overall survival for primary angiosarcoma is approximately 7 months, with 5-year survival rates as low as 10–32%, whereas secondary angiosarcoma shows a median survival of 21–32 months and 5-year survival rates of up to 49% in some series [29,32,33]. Our findings do not support these previously reported differences.

Other studies have investigated whether other cancers besides angiosarcomas were present, without taking into account the temporal relationship to the time of angiosarcoma diagnosis [3].

In this study, women with thoracic angiosarcoma demonstrated a substantially better prognosis than men. The analyses suggest that this survival advantage is not attributable to differences between primary and secondary angiosarcoma or to biological sex per se, but rather to differences in tumor topography and detectability. Almost 80% of angiosarcomas in men were diagnosed in the lung, heart, pleura and mediastinum, whereas these topographies accounted for only about 5% in women. Patients who develop angiosarcoma of the lung, heart, mediastinum, or pleura have a considerably worse prognosis than patients with angiosarcoma of the breast or thoracic skin. This difference may be plausibly explained by earlier detection of breast cancer through screening and follow-up, as well as by the greater accessibility and feasibility of achieving R0 resection for tumors of the breast and thoracic skin. This contrasts with the late detection and high-risk surgical treatment of angiosarcomas of the mediastinum, heart and other thoracic organs or tissues.

Several factors may limit the interpretation of our results. First, as noted in the Introduction, angiosarcoma is associated not only with prior malignancies and their treatments, but also with prior trauma, foreign bodies, exposure to thorium dioxide, and viral infections [9]. Since cancer registry data do not provide information on these risk factors, only prior malignancies were evaluated. The lack of information regarding not only additional risk factors but also angiosarcoma treatment was a limitation of the present study and of most German cancer registries in general.

Second, misclassification of primary and second angiosarcoma is possible due to incomplete cancer registration before 2008. Furthermore, the distinction between primary and second primary angiosarcoma is only an approximate reflection of the underlying clinical reality. Nevertheless, as survival estimates are largely comparable between these groups, this limitation is unlikely to substantially influence our results.

Third, angiosarcoma is an extremely rare diagnosis associated with a poor prognosis. It must be assumed that cases of angiosarcoma of the heart, mediastinum, pleura, or lung are only rarely encountered, even by experienced pathologists. Therefore, misclassification with other vascular malignancies, or even benign lesions, is possible [15].

Fourth, we were unable to include prognostically relevant information on treatment (e.g., R0-surgery, adjuvant radiotherapy or chemotherapy) in the estimation of overall survival, as these data were frequently missing and only available from 2016 onwards. Future studies should investigate the impact of treatment on survival outcomes.

## 5. Conclusions

In conclusion, male patients suffering from thoracic angiosarcoma have a markedly worse prognosis than women. Based on 5-year OS, women with a primary angiosarcoma had a better prognosis than men with a primary angiosarcoma. Contrary to our assumption, the distinction between primary and second primary angiosarcomas played no or only a subordinate role with regard to survival. The topography of the angiosarcoma had the greatest impact on prognosis, with the worst OS was observed for angiosarcoma of the lung, heart, pleura and mediastinum. Finally, these topographies are most frequent in men with thoracic angiosarcoma, whereas they are very rare in women.

## Figures and Tables

**Table 1:** Baseline characteristics of newly diagnosed male and female patients with thoracic angiosarcoma, North Rhine-Westphalia, 2008–2023.

Characteristics	Men		Women	
Angiosarcoma of thorax, morphology 9120/3	42		379	
Mean age of diagnosis (standard deviation)	54.4	(19.8)	70.8	(12.5)
Age at diagnosis (years), *n* (%)				
0–19	0	(0)	1	(0.3%)
20–49	15	(35.7%)	23	(6.1%)
50–59	5	(11.9%)	41	(10.8%)
60–69	11	(26.2%)	76	(20.1%)
70–79	8	(19%)	140	(36.9%)
80+	3	(7.1%)	98	(25.9%)
Topography, *n* (%)				
bronchus and lung (C34)	10	(23.8%)	6	(1.6%)
heart, mediastinum and pleura (C38)	23	(54.8%)	14	(3.7%)
skin of thorax (C44.51)	0	(0%)	33	(8.7%)
soft tissues of thorax (C49.3)	8	(19%)	77	(20.3%)
breast (C50)	1	(2.4%)	249	(65.7%)
Pre-existing diagnosis of skin or breast cancer				
Yes, more than 5 years before AT-diagnosis	0	(0%)	215	(56.7%)
Mean age of diagnosis (standard deviation)			73.1	(10.3)
Yes, up to 5 years before AT-diagnosis	0	(0%)	47	(12.4%)
Mean age of diagnosis (standard deviation)			69.5	(11.6)
No	42	(100%)	117	(30.9%)
Mean age of diagnosis (standard deviation)	54.4	(19.8)	67.0	(15.3)

**Table 2:** 1-, 2- and 5-year survival of patients with thoracic angiosarcoma stratified by status, topography and biological sex in North Rhine-Westphalia, 2008–2023.

Characteristics	*n*	Overall Survival (SE)		
		1 year	2 years	5 years
Overall	421	76.6 (2.1)	62.2 (2.5)	38.5 (2.6)
Men	42	41.2 (7.8)	25.7 (7.0)	12.0 (5.4)
Women	379	80.1 (2.1)	66.3 (2.5)	41.4 (2.8)
Status concerning previous diagnosis of skin/breast cancer				
Men				
Primary	42	41.2 (7.8)	25.7 (7.0)	12.0 (5.4)
Women				
Primary	116	79.2 (3.2)	63.3 (3.9)	40.9 (4.1)
Secondary (0–5 years before)	47	80.0 (6.0)	68.4 (7.0)	44.7 (7.6)
Secondary (>5 years before)	216	80.8 (2.7)	68.7 (3.3)	41.8 (3.8)
Topography				
Overall				
Bronchus and lung (C34)		51.3 (13.3)	22.0 (11.2)	14.7 (9.6)
Heart, mediastinum and pleura (C38)		35.1 (8.1)	14.6 (6.0)	2.9 (2.9)
Skin of thorax (C44.51)		81.8 (6.7)	69.4 (8.1)	34.7 (8.4)
Soft tissues of thorax (C49.3)		78.2 (4.6)	62.9 (5.4)	38.2 (5.9)
Breast (C50)		82.3 (2.5)	70.5 (3.0)	46.1 (3.5)
Men				
Bronchus and lung (C34)		40.0 (15.5)	30.0 (14.5)	20.0 (12.7)
Heart, mediastinum and pleura (C38)		37.3 (10.3)	18.6 (8.4)	4.7 (4.5)
Skin of thorax (C44.51)		-	-	-
Soft tissues of thorax (C49.3)		46.9 (18.7)	31.3 (17.8)	15.6 (14.2)
Breast (C50)		-	-	-
Women				
Bronchus and lung (C34)		80.0 (17.9)	-	-
Heart, mediastinum and pleura (C38)		31.4 (12.9)	7.9 (7.5)	-
Skin of thorax (C44.51)		81.8 (6.7)	69.4 (8.1)	34.7 (8.4)
Soft tissues of thorax (C49.3)		81.3 (4.5)	66.0 (5.5)	40.4 (6.3)
Breast (C50)		82.2 (2.5)	70.3 (3.0)	45.9 (3.5)

## Data Availability

Data is available on request.

## Abbreviations

The following abbreviations are used in this manuscript:

OS	overall survival
NRW	North Rhine-Westphalia
